# *miR-223-3p* predicts prognosis of hepatitis B virus-related acute-on-chronic liver failure and is involved in hepatocyte injury via *HSP90B1*

**DOI:** 10.1186/s41065-025-00610-5

**Published:** 2025-11-29

**Authors:** Feiyue Xie, Qiuping Ren, Jun He, Menghang Wu

**Affiliations:** Department of Liver Surgery, West China Hospital of Sichuan University, No. 37, Guoxue Lane, Wuhou District, Chengdu, 610044 China

**Keywords:** MiR-223-3p, HBV, Liver failure, Prognosis

## Abstract

**Background:**

Hepatitis B virus-related acute-on-chronic liver failure (HBV-ACLF) is a clinical syndrome that presents with acute hepatic decompensation and liver failure in a relatively short time, with a high mortality rate.

**Objective:**

The aim was to assess the predictive value of *miR-223-3p* in the short-term prognosis of patients and its potential role in HBV-ACLF, thus providing new ideas for personalized treatment.

**Materials and methods:**

The level of *miR-223-3p* was quantified using qRT-PCR. The correlation between *miR-223-3p* level and indicators associated with the severity of HBV-ACLF (TBil, INR, and MELD score) was assessed using Spearman’s method. The prognostic value of *miR-223-3p* in HBV-ACLF was assessed using the ROC curve, Cox regression analysis, and Kaplan-Meier curve. To detect the proliferation and apoptosis of MIHA cells, CCK-8 assay and flow cytometry were employed. Bioinformatics methods were conducted to identify the downstream targets of *miR-223-3p*. The regulation between *miR-223-3p* and *HSP90B1* was validated through Dual-luciferase reporter gene assay.

**Results:**

In patients with HBV-ACLF, *miR-223-3p* expression was reduced and negatively correlated with TBil, INR, and MELD score. Low expression of *miR-223-3p* predicted adverse prognosis for patients. Furthermore, MELD score and *miR-223-3p* were identified as independent prognostic factors in patients with HBV-ACLF. In H_2_O_2_ or TNF-α–induced MIHA cells, *miR-223-3p* facilitated cellular proliferation and suppressed apoptosis. The role of *miR-223-3p* in hepatocyte injury was mediated by *HSP90B1*.

**Conclusions:**

Serum *miR-223-3p* expression was predictive for short-term survival in patients with HBV-ACLF, and *miR-223-3p* attenuated hepatocellular injury in vitro by modulating *HSP90B1*.

**Supplementary Information:**

The online version contains supplementary material available at 10.1186/s41065-025-00610-5.

## Introduction

Acute-on-chronic liver failure (ACLF) is a syndrome characterized by the development of coagulation dysfunction and acute jaundice in individuals with chronic liver disease [1]. It can be precipitated by various triggers, including hemorrhage, infection, and other external stimuli [2]. Hepatitis B virus (HBV)-ACLF has the highest prevalence in China and is characterized by complex pathogenesis [3]. Patients may develop complications such as ascites, infection, electrolyte disorders, hepatic encephalopathy, and multi-organ failure. HBV-ACLF is distinguished by its rapid progression and high lethality [4, 5]. A review of clinical studies indicates that the 28-day mortality rate for HBV-ACLF ranges from 25.52% to 48.66%, while the 90-day mortality rate is 50% and higher [6, 7]. Currently, the clinical treatment of patients with HBV-ACLF primarily encompasses etiology, symptomatic support, and the management of complications. Furthermore, the integration of artificial liver support system (ALSS), including plasma exchange, hemofiltration, and plasma adsorption, has the potential to enhance hepatic and renal function, as well as prognosis [8]. The model for end-stage liver disease (MELD) score is valuable for assessing the severity and short-term prognosis of liver disease, but there is a lack of consensus regarding the prognostic value of HBV-ACLF. Accurate prediction of the prognosis of patients with HBV-ACLF is of great significance in guiding the treatment and prolonging the survival time of patients. However, reliable biomarkers to predict prognosis in HBV-ACLF are lacking.

MicroRNAs (miRNAs) constitute a class of small RNAs that usually bind to the 3’ UTR of mRNA to regulate post-transcriptional expression of genes [9]. Recently, many studies have revealed the critical regulatory roles of miRNAs in developmental and pathological processes [10, 11]. For example, Fang et al. elucidated that miR-450b-5p slows the progression of acute liver failure by regulating inflammatory responses and apoptosis in hepatocytes [12]. MiRNAs have been demonstrated to be potentially prognostic biomarkers that could be beneficial for the assessment of diseases in the clinic, including hepatocellular carcinoma, liver failure, and non-alcoholic fatty liver disease [13–15]. Previously, Cisilotto et al. unveiled that *miR-223-3p* was aberrantly expressed in ACLF patients by miRNA microarray analysis [14]. The role of *miR-223-3p* in the pathogenesis and progression of liver diseases has been widely reported [16, 17]. Previous studies have demonstrated that *miR-223-3p* is highly expressed in the liver and hepatocytes [18], and its dysregulation is commonly associated with the pathophysiology of liver diseases such as liver cancer and acute liver injury [19, 20]. *miR-223-3p* was identified as a promising biomarker for the early detection of HBV-ACLF [21]. Nevertheless, the prognostic significance of *miR-223-3p* in HBV-ACLF and the molecular basis of disease progression require further study.

The aim of the research was to analyze the value of *miR-223-3p* in assessing the short-term prognosis of HBV-ACLF, thereby providing a reference for the assessment of patients’ conditions. Additionally, this study evaluated the regulatory role of *miR-223-3p* in HBV-ACLF progression to offer new insights into the molecular mechanism of the disease.

## Materials and methods

### Study subjects

The present study included 122 patients with HBV-ACLF who were diagnosed at West China Hospital of Sichuan University between March 2022 and March 2024. This study was approved by the Institutional Ethics Committee (Approval number: 2022 − 105). Inclusion criteria: (1) the diagnosis of HBV-ACLF following the guideline for diagnosis and treatment of liver Failure (2018) [22]; and (2) complete clinical data. Exclusion criteria: (1) liver disease resulting from alcohol use, genetic predisposition, metabolic disorders, drug toxicity, fatty liver disease, or autoimmune conditions; (2) co-infection with other hepatitis viruses; (3) co-existence of malignant neoplasms and systemic hematologic disorders; (4) congenital heart disease; and (5) pregnancy or lactation. A control group of 50 patients with chronic hepatitis B (CHB) who had been excluded from the liver failure cohort was also included in the same period. Baseline data, including age and gender, were collected for study subjects at the time of admission. Test indicators were collected within 24 h of admission and included routine blood tests [platelet count (PLT), white blood cells (WBC)], liver function tests [alanine aminotransferase (ALT), total bilirubin (TBil), albumin, aspartate aminotransferase (AST)], serum creatinine (SCr), and coagulation function tests [international normalized ratio (INR)]. The MELD score was calculated according to the formula: MELD score = 9. 6 × ln [Cr (mg/dL)] + 3. 8 × ln [TBil (mg/dL)] + 11. 2 × ln (INR) + 6. 4 × etiology score (cholestatic or alcoholic is 0, and other causes such as viruses are 1) [23]. A total of 5 mL of venous blood was obtained from each subject and subsequently centrifuged at 3,000 rpm for 15 min. The supernatant was then stored at −80 °C for subsequent analysis.

### Follow-up survey

All patients with HBV-ACLF were provided with the same comprehensive therapeutic regimen, including artificial liver support if necessary. The 28-day follow-up results of patients were used to categorize them into survival or death groups: the former group included 82 cases, while the latter comprised 40 cases.

### Cell lines, transfection, and treatment

Normal human liver cells (MIHA, ATCC, USA) were cultured in RPMI 1640 medium (Cat# 11875093, Gibco, USA) supplemented with 1% penicillin-streptomycin (Cat# P1400, Solarbio, Beijing, China), 10% fetal bovine serum (FBS) (Cat# 10099158, Gibco). The *miR-223-3p* mimic (Cat# HY-R04313), inhibitor (Cat# HY-RI00467), and negative control (miR NC) bought from MedChemExpress (USA) were transfected into cells with Lipofectamine 3000 Reagent (Cat# L3000001, Thermo Fisher Scientific, USA). The pcDNA3.1 vector (Cat# V79520, Thermo Fisher Scientific) was employed for the construction of the *HSP90B1* overexpression plasmid (*HSP90B1*-oe). To induce hepatocyte injury, MIHA cells were exposed to either 0.1 mmol/L H₂O₂ for 3 h or 20 ng/mL TNF-α for 24 hours.

### RNA extraction and qRT-PCR

RNA was extracted from serum and cells by TRIzol reagent (Cat# 15596018CN, Invitrogen, USA), as previously described. The concentration and quality of the RNA were assessed using a NanoDrop spectrophotometer (Thermo Fisher Scientific), with A_260_/A_280_ ratios approximately equal to 2.0. The RNA was reverse transcribed into cDNA using the Reverse Transcription Kit (Cat# 205311, Qiagen, Germany). The qPCR system was prepared in accordance with the instructions provided by SYBR Premix ExTaq (Cat# RR041A, TaKaRa, Japan) with three replicates, and *U6*/*GAPDH* was used as endogenous controls. The reaction was conducted on a CFX96 qRT-PCR instrument (BIO-RAD, USA), and the reaction conditions were set as follows: step 1, 95 °C for 30 s; step 2, 40 cycles of 95 °C for 5 s and 60 °C for 30 s. The sequences of all primers are provided in Supplementary Materials (supplementary materials, Table S1). The relative level of serum *miR-223-3p* (UGUCAGUUUGUCAAAUACCCCA) and *HSP90B1* mRNA (RefSeq: NM_003299.3) was calculated using the 2^−∆∆Ct^ method. Each sample was analyzed in triplicate, and experiments were repeated independently at least three times.

### CCK-8 assay

Group-treated MIHA cells were inoculated into 96-well culture plates, and cell viability was determined by adding 10 µL of CCK-8 reagent (reference incubation time: 1–4 h; Cat# CK04, Dojindo, Japan) to each well. The absorbance was assessed at 450 nm using Infinite M200 Multifunctional Enzyme Labeler (Tecan, Switzerland) following a 2-hour incubation period at 37 °C. All experiments were performed in triplicate. This experimental protocol was based on methods employed in previous studies [24, 25]. All detected absorbance values fall within the specified linear range.

### Apoptosis assay

Cell apoptosis was evaluated using FITC/Annexin V/Apoptosis Detection Kit (Cat# BMS500FI-100, Invitrogen). Briefly, group-treated cells were resuspended in 1 × Annexin binding buffer. The reaction was carried out by adding 5 µL of Annexin V-Fluorescein isothiocyanate solution and 1 µL of propidium iodide for 15 min and protected from light. A total of 400 µL of 1 × Annexin binding buffer was added and gently mixed. Apoptosis was analyzed within 1 h using FACSCanto II flow cytometer (BD Biosciences, USA).

### Bioinformatics analysis

The online databases TargetScanHuman (https://www.targetscan.org/vert_72/), miRDB (https://mirdb.org/cgi-bin/search.cgi), miRWalk (http://mirwalk.umm.uni-heidelberg.de/), starBase (https://rnasysu.com/encori/), and TarBase (https://dianalab.e-ce.uth.gr/tarbasev9) predicted the targets of *miR-223-3p*. A Venn diagram was constructed using the bioinformatics online platform (https://www.bioinformatics.com.cn/). The TargetScanHuman database was employed to predict the binding sites of *miR-223-3p* and *HSP90B1*.

### Dual-luciferase reporter gene assay

The luciferase reporter plasmids *HSP90B1*-wild type (WT) and *HSP90B1*-mutant (MUT) were constructed based on the predicted sites to *miR-223-3p*. The luciferase reporter vector was co-transfected with *miR-223-3p* mimic, inhibitor, or miR NC in MIHA cells. Cells were lysed to detect luciferase activity using the Dual-Luciferase Reporter Assay (Cat# E1910, Promega, USA).

### RNA Immunoprecipitation (RIP)

RIP was conducted in MIHA cells with the Magna RIP kit (Cat# 17–700, Sigma-Aldrich, USA) following the manufacturer’s instructions. Transfected cells (*miR-223-3p* or miR NC) were lysed in RIP lysis buffer and incubated with magnetic beads conjugated to anti-Ago2 antibody or normal IgG. The enrichment of *HSP90B1* mRNA was quantified by qRT-PCR.

### Western blot

Proteins were extracted from cultured cells using RIPA lysis buffer (Cat# 89900, Thermo Fisher Scientific). After quantification with the BCA assay, equal amounts of protein were separated by SDS-PAGE and transferred to PVDF membranes. The membranes were blocked with 5% non-fat milk and incubated overnight at 4 °C with primary antibody against HSP90B1 (1:1000, Cat# ab238126, Abcam, UK), followed by incubation with an HRP-conjugated secondary antibody (1:5000, Cat# ab205718, Abcam). Protein bands were visualized using BeyoECL SuperMoon (Cat# P0018HS, Beyotime, China), and band intensities were quantified using ImageJ software.

### Statistical analysis

SPSS 23.0 was applied for statistics, and GraphPad Prism 9.0 was employed to visualize the results. Measurements that were normally distributed were expressed as mean ± standard deviation and assessed using t-test or ANOVA. Data not normally distributed were expressed as median (25%, 75% interquartile range), and comparisons were performed using the Mann-Whitney U test. Correlation analysis between *miR-223-3p* and clinical indicators was performed using Spearman’s method. Count data were analyzed by Chi-square test. Kaplan-Meier curve, ROC curve, and Cox regression were applied to evaluate the short-term prognostic value of *miR-223-3p* in HBV-ACLF patients. *P* < 0.05 was considered a statistically significant difference.

## Results

### miR-223-3p was related to HBV-ACLF

**Fig. 1:**
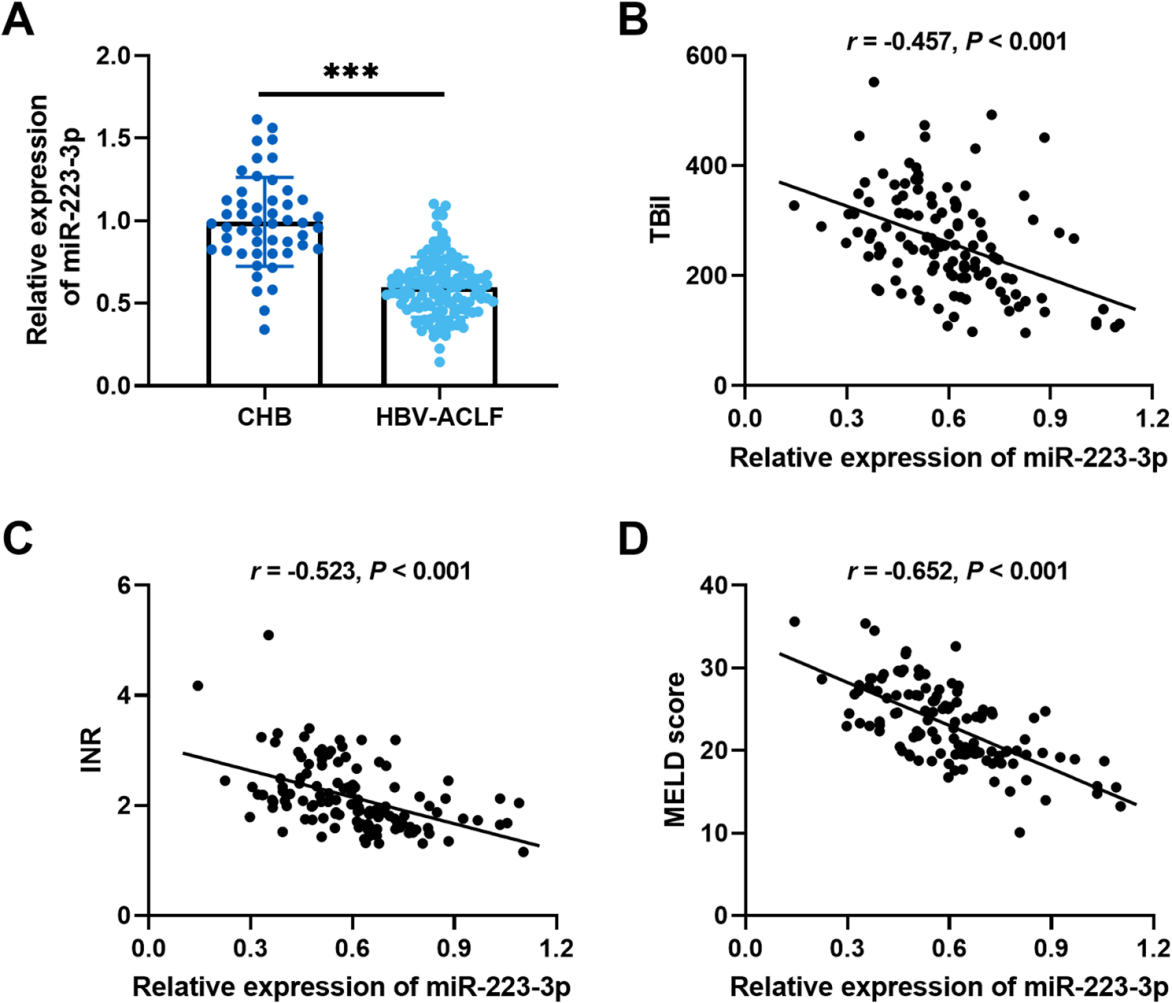
Association of *miR-223-3p* with HBV-ACLF. **A.** The levels of *miR-223-3p* in patients with CHB and patients with HBV-ACLF (Student’s t-test, ***P < 0.001). **B-D.** Correlation of *miR-223-3p* with clinical indicators TBil (**B**), INR (**C**), and MELD score (**D**) in patients with HBV-ACLF. (nonparametric Spearman test, *P* < 0.05)

There were 122 patients with HBV-ACLF and 50 patients with CHB (controls). The baseline data indicated that patients in the HBV-ACLF group exhibited significantly elevated levels of ALT, TBil, AST, INR, and Scr in comparison to the CHB group, while albumin and PLT levels were reduced (Table 1). The level of *miR-223-3p* was decreased in HBV-ACLF patients compared with controls (Fig. 1A). Furthermore, the level of *miR-223-3p* negatively correlated with TBil (*r* = −0.457, *P* < 0.001, Fig. 1B), INR (*r* = −0.523, *P* < 0.001, Fig. 1C), and MELD score (*r* = −0.652, *P* < 0.001, Fig. 1D).

**Table 1:** Baseline characteristics of the study population

Characteristic	HBV-ACLF group (*n* = 122)	CHB group (*n* = 50)	*P* value	Cohen’s d or OR (95% CI)
Age (years)	53 (37, 67)	55 (37.75, 68.25)	0.500	0.117 (−0.212, 0.447)
Gender, male (%)	85 (69.67)	33 (66.00)	0.637	0.845 (0.419, 1.703)
WBC (× 10^9^/L)	7.17 (5.17, 9.70)	6.27 (4.88, 8.80)	0.241	0.194 (−0.136, 0.524)
ALT (U/L)	156.25 (102.18, 213.63)	61.25 (46.25, 74.88)	< 0.001	2.345 (1.921, 2.765)
AST (U/L)	166.15 (139.53, 191.95)	75.60 (52.55, 92.48)	< 0.001	2.317 (1.903, 2.727)
TBil (µmol/L)	254.80 (195.40, 317.08)	36.50 (28.80, 51.30)	< 0.001	4.292 (3.677, 4.896)
Albumin (g/L)	31.60 (26.50, 40.00)	37.20 (34.55, 40.83)	< 0.001	−0.666 (−1.002, −0.328)
INR	2.02 (1.73, 2.42)	1.04 (0.94, 1.18)	< 0.001	2.962 (2.495, 3.425)
Scr (µmol/L)	78.45 (63.48, 93.43)	67.35 (59.13, 85.48)	0.012	0.516 (0.179, 0.850)
PLT (× 10^9^/L)	86.00 (62.75, 120.25)	187.50 (144.00, 229.50)	< 0.001	−1.725 (−2.164, −1.279)
MELD score	22.97 (19.59, 26.66)	-	-	-

Quantitative variables were presented as median (25%, 75% interquartile range). Categorical variables were expressed as number (%)

*Abbreviations*: *HBV-ACLF* hepatitis B virus-related acute-on-chronic liver failure, *CHB* chronic hepatitis B, *CI* confidence interval, *OR* odds ratio, *WBC* white blood cell, *ALT* alanine aminotransferase, *AST *aspartate aminotransferase, *TBil* total bilirubin, *INR *international normalized ratio, *Scr* serum creatinine, *PLT* platelet count, *MELD* model for end-stage liver disease

### miR-223-3p could predict the prognosis of HBV-ACLF patients

**Fig. 2:**
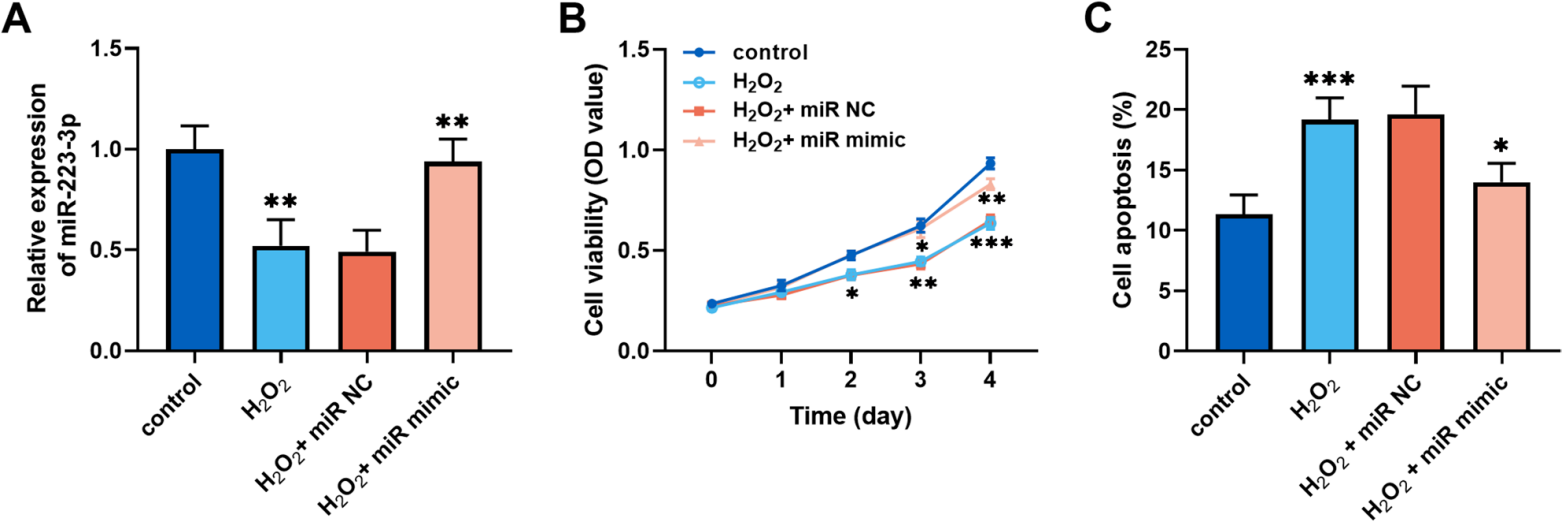
Aberrantly expressed *miR-223-3p* predicted poor prognosis of HBV-ACLF patients. **A.** *miR-223-3p* levels in HBV-ACLF patients with different survival status (Student’s t-test, ***P < 0.001). **B.** Reduced expression of *miR-223-3p* was related to low survival in HBV-ACLF patients (log-rank test, *P* < 0.001). **C-E.** ROC curves for *miR-223-3p* (**C**), MELD score (**D**), and their combination (**E**) in distinguishing HBV-ACLF patients with different prognostic statuses. (Mann-Whitney U test, *P* < 0.001)

All patients were followed for 28 days, while 40 patients died, resulting in the case-fatality rate of 32.79%. The clinical data revealed elevated levels of TBil, INR, Scr, and MELD score in the patients who died. Furthermore, a greater proportion of patients who died experienced complications and organ failure compared to survivors. (Table 2) The level of *miR-223-3p* was decreased in deceased patients in comparison to surviving patients with HBV-ACLF (Fig. 2A). Based on the mean *miR-223-3p* level, patients with HBV-ACLF were stratified into two groups for survival analysis: the high *miR-223-3p* level group and the low *miR-223-3p* level group. The findings indicated that patients with low *miR-223-3p* levels exhibited a higher mortality than those with high *miR-223-3p* levels (Fig. 2B). Furthermore, Cox regression analysis identified *miR-223-3p* (HR = 0.197, 95% CI = 0.090–0.432, *P* < 0.001) and MELD score (HR = 3.695, 95% CI = 1.023–13.347, *P* = 0.046) as independent prognostic factors of HBV-ACLF (Table 3). Cox regression analysis revealed no significant interaction between *miR-223-3p* and the MELD score (*P* = 0.132). Prior to development of the Cox model, multicollinearity assessment was performed for the MELD score and its constituent variables (TBil, INR, and Scr) using tolerance (Tol) and variance inflation factor (VIF) diagnostics. The analysis demonstrated acceptable levels of multicollinearity for all parameters: MELD score (VIF = 2.722, Tol = 0.367), TBil (VIF = 1.324, Tol = 0.755), INR (VIF = 1.678, Tol = 0.596), and Scr (VIF = 1.547, Tol = 0.646). All VIF values were well below the conservative threshold of 5, and all Tol values exceeded 0.1, confirming the absence of significant multicollinearity that could compromise the regression model. Furthermore, subgroup analyses stratified by age, gender, and ACLF grade further underscored the association between *miR-223-3p* and HBV-ACLF prognosis (supplementary materials, Table S2).

**Table 2:** Clinical information of patients in the survival and death groups

Characteristic	survival group (*n* = 82)	death group (*n* = 40)	*P* value	Cohen’s d or OR (95% CI)
Age (years)	52.5 (36, 65.25)	56.50 (41, 69.75)	0.164	0.265 (−0.115, 0.644)
Gender, male (%)	55 (67.07)	30 (75.00)	0.371	0.679 (0.290, 1.590)
WBC (× 10^9^/L)	7.08 (4.76, 9.60)	7.57 (5.51, 9.90)	0.395	0.230 (−0.150, 0.609)
ALT (U/L)	152.55 (95.98, 218.03)	162.65 (119.33, 202.48)	0.416	0.192 (−0.188, 0.570)
AST (U/L)	160.70 (140.20, 189.95)	179.15 (138.88, 193.35)	0.301	0.007 (−0.301, 0.308)
TBil (µmol/L)	227.80 (167.50, 305.23)	292.35 (247.13, 343.70)	0.001	0.652 (0.263, 1.037)
Albumin (g/L)	31.45 (25.08, 38.98)	31.80 (27.75, 41.23)	0.560	−0.071 (−0.449, 0.307)
INR	1.89 (1.64, 2.25)	2.30 (2.01, 2.95)	< 0.001	0.753 (0.348, 1.150)
Scr (µmol/L)	70.75 (60.23, 82.95)	93.30 (80.38, 114.18)	< 0.001	1.266 (0.854, 1.675)
PLT (× 10^9^/L)	91.00 (68.00, 127.75)	76.50 (59.75, 117.50)	0.088	−0.344 (−0.724, 0.037)
Ascites, n (%)	48 (58.54)	31 (77.50)	0.040	2.440 (1.030, 5.780)
Hepatic encephalopathy, n (%)	12 (14.63)	23 (57.50)	< 0.001	7.892 (3.286, 18.958)
Gastrointestinal hemorrhage, n (%)	1 (1.22)	6 (15.00)	0.002	14.294 (1.657, 123.273)
Renal failure, n (%)	2 (2.44)	5 (12.50)	0.025	5.714 (1.057, 30.883)
Infection, n (%)	42 (51.22)	28 (70.00)	0.051	2.222 (0.996, 4.961)
ACLF grade III, n (%)	11 (13.41)	16 (40.00)	0.001	4.303 (1.756, 10.545)
MELD score	19.95 (18.78, 23.99)	26.97 (24.47, 28.76)	< 0.001	1.573 (1.143, 1.998)

Quantitative variables were presented as median (25%, 75% interquartile range). Categorical variables were expressed as number (%)

*Abbreviations*:* CI* confidence interval, *OR *odds ratio, *WBC* white blood cell, *ALT* alanine aminotransferase, *AST* aspartate aminotransferase, *TBil* total bilirubin, *INR* international normalized ratio, *Scr *serum creatinine, *PLT* platelet count, *ACLF* acute-on-chronic liver failure, *MELD* model for end-stage liver disease

**Table 3:** Association of clinical characteristics with overall survival of patients

Characteristic	HR factor	95% CI	*P* value
Age	1.087	0.567–2.086	0.801
Gender	0.611	0.271–1.380	0.236
TBil	1.734	0.825–3.647	0.147
INR	2.264	0.939–5.455	0.069
Scr	1.030	0.377–2.809	0.955
Infection	1.339	0.628–2.854	0.450
ACLF grade	1.650	0.828–3.287	0.155
MELD score	3.695	1.023–13.347	0.046
*miR-223-3p*	0.197	0.090–0.432	< 0.001

*Abbreviations*: *HR* hazard ratio,* CI* confidence interval, *TBil* total bilirubin, *INR* international normalized ratio, *Scr* serum creatinine, *ACLF* acute-on-chronic liver failure, *MELD* model for end-stage liver disease

The ROC curve showed that the AUC value, sensitivity, and specificity of *miR-223-3p* to distinguish different survival states were 0.875 [95% confidence interval (CI): 0.807–0.944], 72.50%, and 92.68%, respectively (Fig. 2C). The AUC of the ROC curve for the MELD score was 0.867 (95% CI: 0.805–0.927), with sensitivity and specificity of 95.00% and 67.07%, respectively (Fig. 2D). The results of the DeLong test comparing the two models were not statistically significant (*P* = 0.841). However, *miR-223-3p* demonstrated higher specificity in distinguishing patient survival status. Furthermore, the ROC analysis combining *miR-223-3p* with the MELD score yielded an AUC of 0.917 (95% CI: 0.866–0.967), with sensitivity and specificity of 90.00% and 81.71%, respectively (Fig. 2E). The DeLong test revealed that combining *miR-223-3p* with MELD score provided significantly better ROC performance than either marker alone (*P* = 0.034 and *P* = 0.038, respectively).

### miR-223-3p affected cellular behavior of hepatocytes

**Fig. 3:**
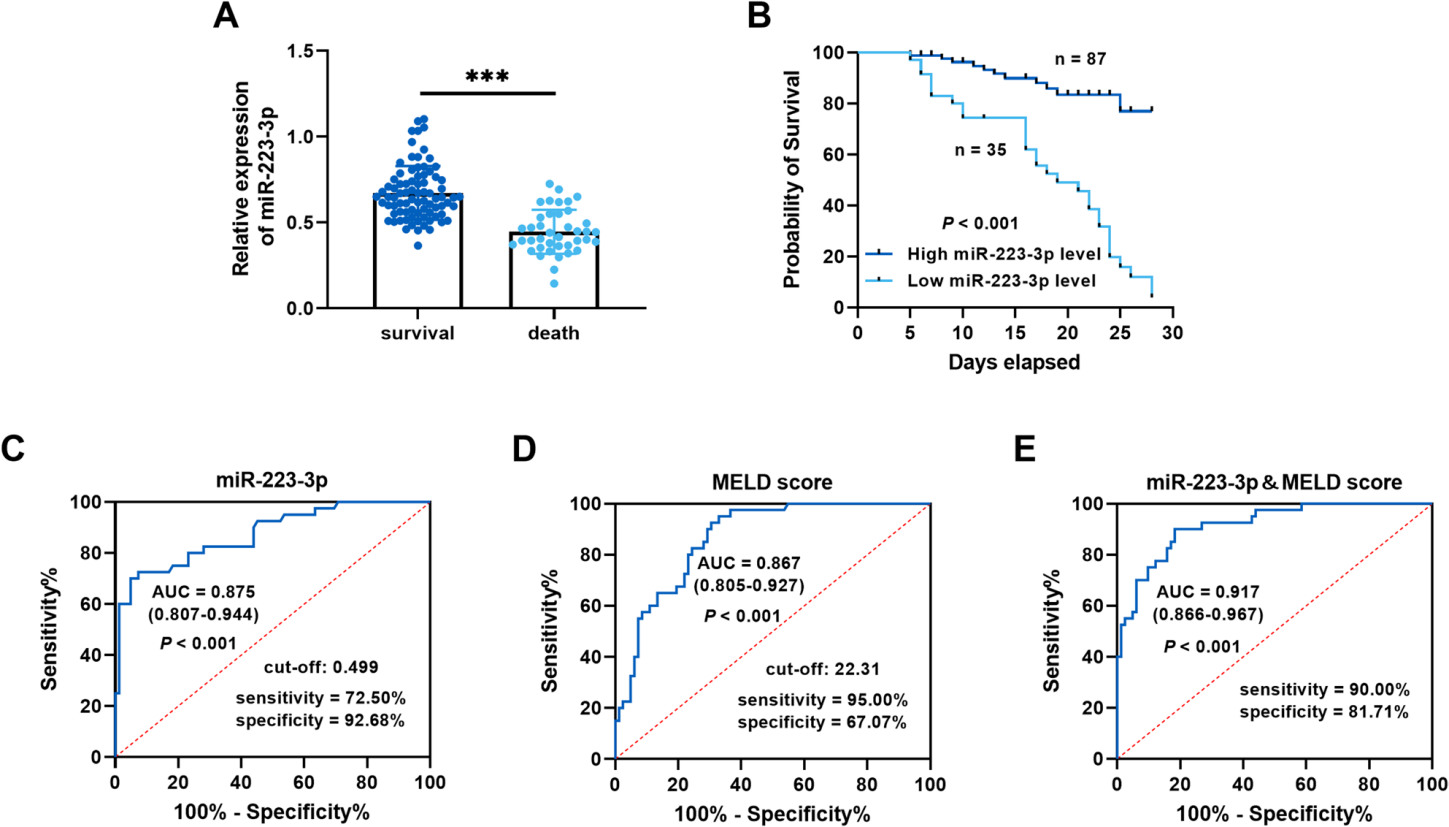
Overexpression of *miR-223-3p* attenuated H_2_O_2_-induced MIHA cell injury. **A.** H_2_O_2_ downregulated *miR-223-3p* expression in MIHA cells, whereas transfection of *miR-223-3p* mimic increased *miR-223-3p* levels. **B-C.** In H_2_O_2_-induced MIHA cells, *miR-223-3p* mimic promoted cell proliferation (**B**) and inhibited apoptosis (**C)** . (one-way or two-way ANOVA with Turkey’s test, *P < 0.05, **P < 0.01, ***P < 0.001)

To elucidate the impact of *miR-223-3p* on hepatocyte injury, MIHA cells were treated with H₂O₂ for experimental purposes. In H_2_O_2_-induced MIHA cells, *miR-223-3p* was markedly downregulated, whereas *miR-223-3p* mimic markedly elevated *miR-223-3p* levels (Fig. 3A). *miR-223-3p* mimic promoted the proliferation and inhibited apoptosis of H_2_O_2_-induced MIHA cells (Fig. 3B and C).

### HSP90B1 was a target of miR-223-3p

**Fig. 4:**
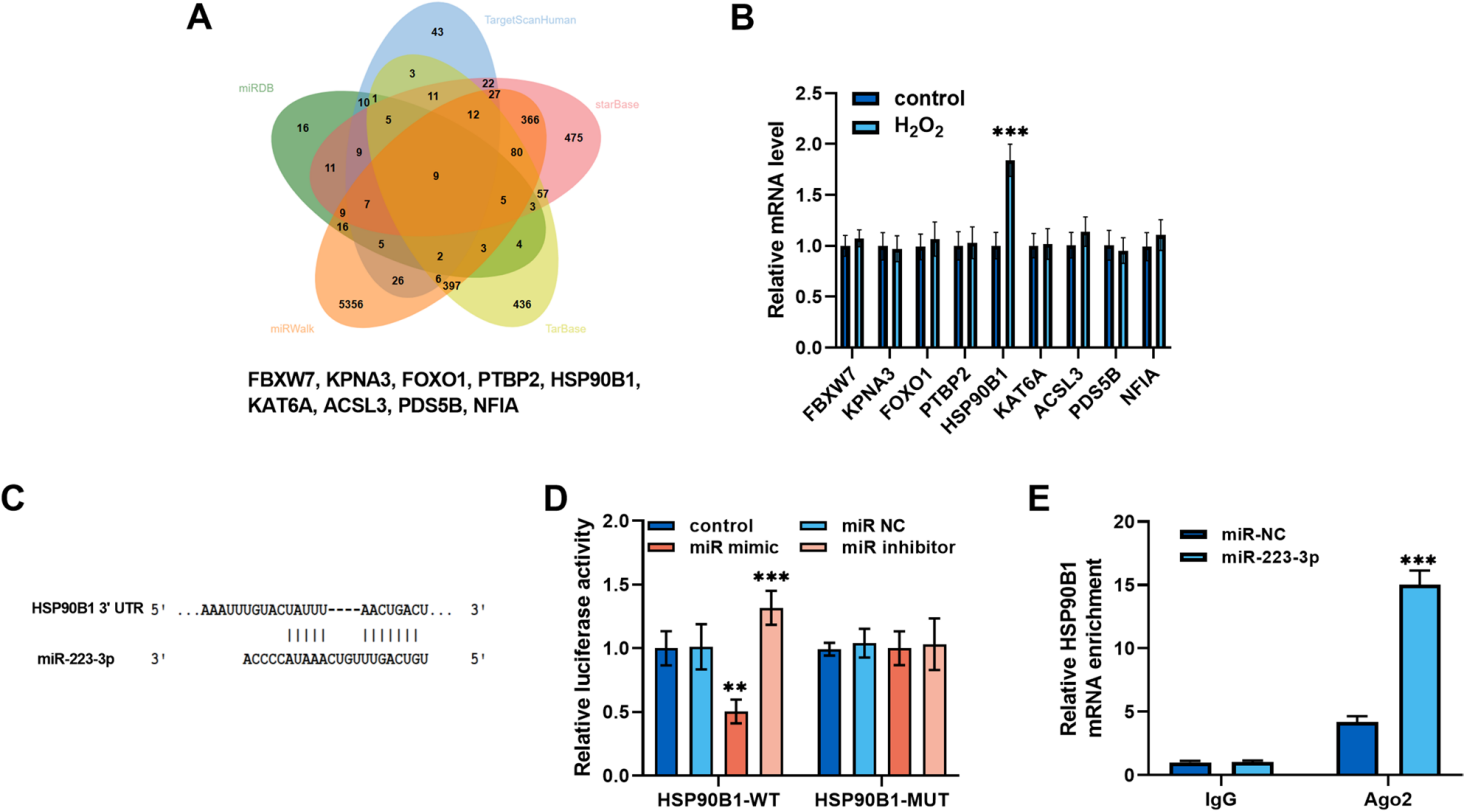
*HSP90B1* was the downstream gene of *miR-223-3p*. **A.** Online databases TargetScanHuman, miRDB, miRWalk, starBase, and TarBase predicted targets of *miR-223-3p*. **B.** *HSP90B1* expression was upregulated in H_2_O_2_-induced MIHA cells. **C.** Potential binding sites of *miR-223-3p* to *HSP90B1*. **D.** Dual-luciferase reporter gene assay validated the targeting relationship between *miR-223-3p* and *HSP90B1.* **E.** RIP assays were performed using anti-Ago2 or IgG antibodies in MIHA cells, followed by qRT-PCR to detect *HSP90B1* mRNA enrichment. (one-way ANOVA with Turkey’s test, **P < 0.01, ***P < 0.001)

**Fig. 5:**
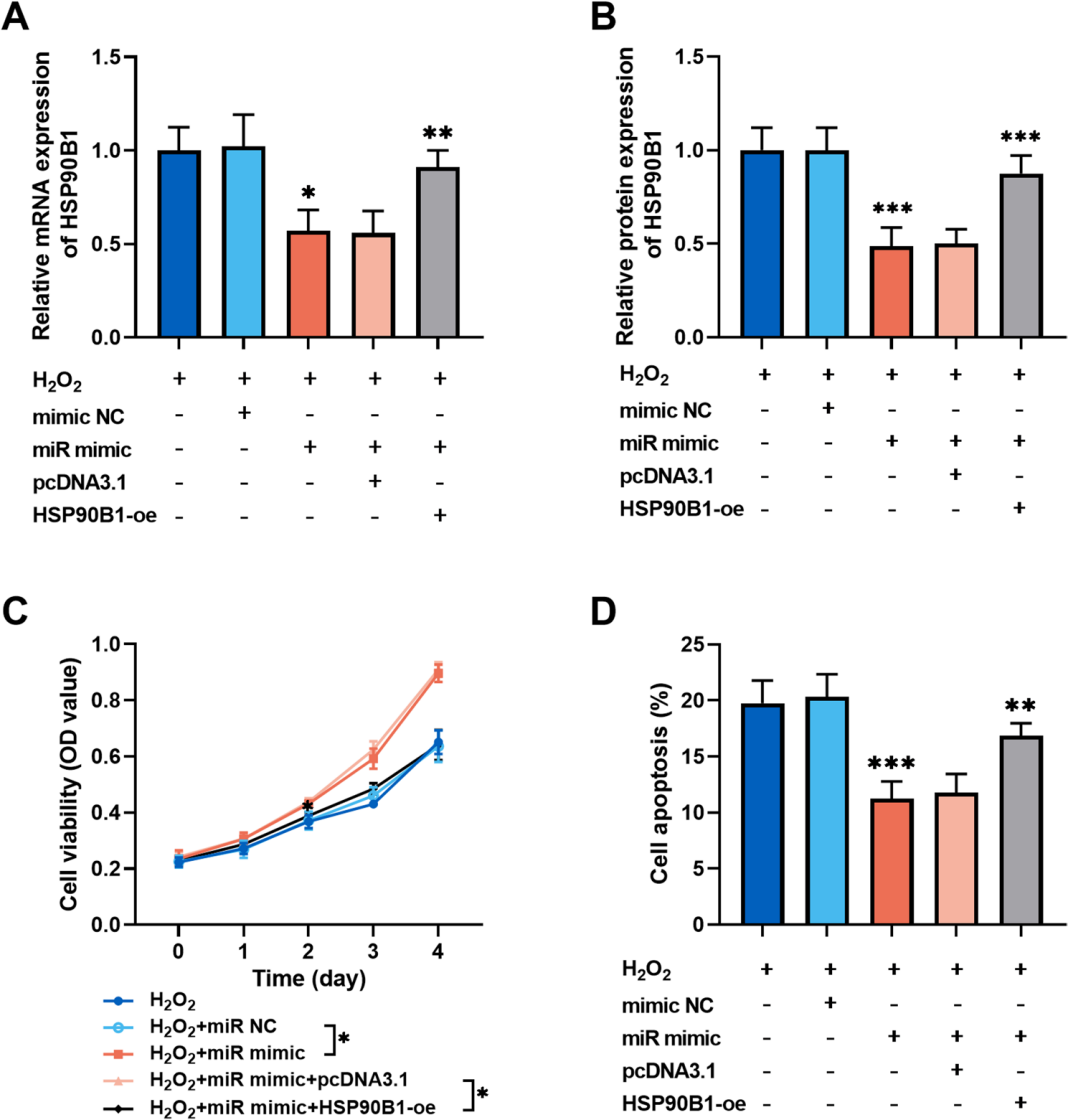
The biological functions of *miR-223-3p* were exerted through the regulation of *HSP90B1*. **A-B.** *miR-223-3p* suppressed *HSP90B1* mRNA (**A**) and protein (**B**) expression, whereas transfection of *HSP90B1*-oe restored its expression. **C-D.** In H_2_O_2_-induced MIHA cells, overexpression of *HSP90B1* repressed the promotion of cell proliferation (**C**) and attenuated the inhibition of apoptosis (**D**) by *miR-223-3p.* (one-way or two-way ANOVA with Turkey’s test, *P < 0.05, **P < 0.01, ***P < 0.001)

We also explored the molecular mechanisms in vitro. The downstream targets of *miR-223-3p* were predicted by TargetScanHuman, miRDB, miRWalk, starBase, and TarBase databases, resulting in the identification of nine potential genes (Fig. 4A). Among the genes examined, only *HSP90B1* demonstrated elevated expression in H₂O₂-induced MIHA cells (Fig. 4B). The binding sites of *miR-223-3p* and *HSP90B1* were predicted with TargetScanHuman database (Fig. 4C). *miR-223-3p* overexpression remarkably reduced the luciferase activity of *HSP90B1*, whereas the inhibition of *miR-223-3p* increased it (Fig. 4D). Enrichment of *HSP90B1* mRNA was detected via RIP analysis with Ago2 antibody (Fig. 4E). To further reveal the molecular mechanism of *miR-223-3p* in hepatocyte injury, co-transfection experiments were conducted. The *HSP90B1* overexpression vector significantly increased *HSP90B1* mRNA expression (supplementary materials, Figure S1). In H_2_O_2_-induced MIHA cells, *miR-223-3p* mimic suppressed *HSP90B1* expression, whereas the *HSP90B1* overexpression vector increased the mRNA and protein levels of *HSP90B1* (Fig. 5A and B). In H_2_O_2_-induced hepatocytes, overexpression of *HSP90B1* exhibited inhibitory effect on the promotion of cell growth by *miR-223-3p* (Fig. 5C**).** The reverse effect of *HSP90B1* was also observed in the inhibition of apoptosis by *miR-223-3p* (Fig. 5D**)**. To further investigate the role of *miR-223-3p* in inflammatory injury of hepatic cells, MIHA cells were stimulated with TNF-α. In this model, *miR-223-3p* expression was markedly downregulated, an effect that was rescued by transfection with *miR-223-3p* mimic (supplementary materials, Figure S2A). Upregulation of *miR-223-3p* also counteracted the TNF-α-induced elevation in *HSP90B1* expression. Conversely, co-transfection with *HSP90B1* overexpression vector restored *HSP90B1* mRNA levels (Figure S2B). Functional assays showed that *miR-223-3p* alleviated TNF-α-induced reduction in cell viability and inhibited apoptosis, while overexpression of *HSP90B1* reversed these protective effects (Figure S2C and D).

## Discussion

HBV-ACLF progresses rapidly and has a high short-term mortality rate. To date, no effective treatment for this disease has been identified [26]. Recently, the continuous development of ALSS technology in the clinical treatment of chronic liver disease has contributed to a reduction in the mortality rate of patients with HBV-ACLF. However, the efficacy of this approach remains a topic of debate [27, 28]. The early assessment of the short-term prognosis of HBV-ACLF based on relevant indicators is a key step in the clinical development and implementation of prevention and treatment strategies [4, 29]. It is therefore important to actively explore simple and accurate indicators to predict the prognosis of HBV-ACLF. This study focused on the clinical significance of *miR-223-3p* in HBV-ACLF and conducted a preliminary exploration of its role in hepatocellular injury.

Hepatic damage caused by HBV-ACLF results in a significant decline in liver function, leading to impaired synthesis, detoxification, biotransformation, and excretion processes [30]. Notably, infection, coagulation dysfunction, and increased bilirubin represent significant clinical features of patients with HBV-ACLF [31, 32]. Alterations in TBil and INR reflect the number and function of residual hepatocytes, as well as the reserve function to a certain extent [33, 34]. There were significant alterations in the indicators of liver function, coagulation function, and infection in patients with HBV-ACLF. It has been demonstrated that *miR-223-3p* exerts a regulatory effect on both acute and chronic liver injury processes [20, 35]. This study demonstrated that *miR-223-3p* was markedly downregulated in patients with HBV-ACLF in comparison to patients with CHB, which is consistent with previous results [14]. Here, *miR-223-3p* negatively correlated with TBil, INR, and MELD score in patients. This indicates that the aberrant expression of *miR-223-3p* is related to the severity and progression of HBV-ACLF.

The involvement of miRNAs in HBV-ACLF pathological mechanisms has been reported, such as *miR-218-5p* and *miR-124* [36, 37]. Previously, *miR-223-3p* was reported to have the potential to predict patient prognosis as a biomarker for bronchiectasis, lung cancer, and HBV-related hepatocellular carcinoma [38–40]. Herein, the value of *miR-223-3p* in the short-term prognosis of HBV-ACLF patients was further analyzed. Following a 28-day follow-up, 40 out of 122 patients with HBV-ACLF died, with a mortality rate of 32.79%. Findings from other clinical studies indicated that the 28-day mortality rate of HBV-ACLF patients ranged from 25.1% to 40.6% [41, 42]. This illustrates the rapid progression of the disease in patients with HBV-ACLF. Low *miR-223-3p* expression in patients with HBV-ACLF was linked to lower survival. Previous studies have identified *miR-25-3p* as being associated with 30-day mortality in patients with ACLF [14]. Other research has reported elevated levels of *miR-6840-3p* and *miR-6861-3p* in PBMCs from HBV-ACLF patients, with AUC values of 0.665 and 0.700, respectively, for predicting prognosis [43]. This study demonstrates that *miR-223-3p* exhibits superior prognostic performance (AUC: 0.875) with favorable sensitivity and specificity, suggesting its potential clinical utility. Clinical value analysis indicated that combining *miR-223-3p* with the MELD score enhances predictive accuracy, offering a foundation for future clinical translation. Moreover, *miR-223-3p* was identified as an independent predictor of short-term prognosis in patients with HBV-ACLF. This study is the first to systematically evaluate the prognostic value of *miR-223-3p* in HBV-ACLF. These findings provide further evidence of the clinical significance of *miR-223-3p*, which may inform clinicians’ guidance on individualized treatment. Recent evidence has indicated that other miRNAs, such as *miR-218-5p* and *miR-124*, are also associated with ACLF prognosis [36, 37]. However, as the expression levels of these miRNAs were not measured in the present study, direct comparisons could not be performed. Future investigations should include simultaneous measurement of multiple miRNAs within the same cohort to elucidate the dominant or complementary role of *miR-223-3p*.

Liver injury is characterized by the death or functional impairment of liver cells. Severe or persistent liver injury ultimately results in liver failure, which is a stage of abrupt deterioration of liver function [44]. The present study investigated the regulatory role of *miR-223-3p* on hepatocyte injury. In H_2_O_2_ or TNF-α–induced MIHA cells, *miR-223-3p* enhanced cell growth and inhibited apoptosis, indicating its protective role in hepatocyte injury. Although such cell models are not capable of fully simulating the virus-specific components and inflammatory cytokine storms involved in HBV-ACLF, these experiments demonstrated the protective effect of *miR-223-3p* against hepatocyte damage. While preliminary results from this model provide valuable information about the biological functions of *miR-223-3p*, future studies should use more clinically relevant systems, such as HBV-replicating hepatocytes (e.g., HBV-infected HepG2-NTCP cells) or cytokine cocktails, to validate these findings and improve clinical relevance to HBV-ACLF.

miRNAs are involved in disease progression, usually by regulating mRNAs and thus affecting protein expression [45]. Previous research has indicated that *miR-223-3p* modulates the growth and aggressiveness of bladder cancer cells through *HSP90B1* [46]. This study revealed that *HSP90B1* expression was upregulated in H_2_O_2_ or TNF-α–induced MIHA cells and was negatively modulated by *miR-223-3p*. It has been reported that *HSP90B1* expression is upregulated during liver injury and is involved in processes such as apoptosis and inflammation [47]. *HSP90B1* (GP96) functions as a molecular chaperone essential for the proper folding and membrane localization of Toll-like receptors (TLRs), including TLR2 and TLR4. Its deficiency disrupts TLR signaling, as evidenced in macrophages, where *HSP90B1* knockdown impairs TLR4-mediated activation of the MyD88/TRIF pathway, leading to suppressed NF-κB nuclear translocation and reduced secretion of proinflammatory cytokines [48, 49]. Research indicates that hepatitis C virus infection induces TGF-β1 secretion via *HSP90B1* (GRP94)-mediated NF-κB activation, contributing to liver fibrosis pathogenesis [50]. Additionally, trinitrotoluene upregulates *HSP90B1* (GRP94) and the pro-apoptotic factor CHOP in HepG2 cells, likely via endoplasmic reticulum stress pathway activation, ultimately promoting apoptosis [51]. While prior research established the regulatory role of *miR-223-3p* through E2F1 [16] and PDL1 [17] in liver disease, this work is the first to identify *HSP90B1* as a novel target mediating hepatocyte apoptosis in HBV-ACLF, significantly expanding the understanding of the molecular mechanisms of *miR-223-3p*.The current study further discovered that *miR-223-3p* attenuated liver injury by modulating the proliferation and apoptosis of damaged hepatocytes through *HSP90B1*. Therefore, *miR-223-3p* may participate in the progression of HBV–ACLF through regulation of *HSP90B1*, providing initial mechanistic insight into its biological function. These findings are potentially useful for the clinical treatment of HBV-ACLF. The present study mainly focused on the upstream regulatory mechanism of *HSP90B1*, while its downstream signaling pathways (e.g., NF-κB, JNK, etc.) have not yet been explored experimentally in depth. Notably, based on the results of this study and previous literature, we hypothesized that the endoplasmic reticulum stress pathway may be an important downstream mechanism. It has been shown that the functional inhibition of *HSP90B1*, an endoplasmic reticulum chaperone protein, is closely related to the activation of apoptosis-related molecules, such as CHOP [51]. Therefore, comprehensively elucidating how *miR-223-3p*/*HSP90B1* precisely regulates specific signaling pathways, such as ER stress and NF-κB, and ultimately leads to the alteration of cell fate, will be the direction of our focused research in the future.

### Limitations

The study is susceptible to potential selection bias. As a single-center study, our cohort may not be fully representative of the broader HBV-ACLF population. This could lead to an overestimation of the prognostic efficacy of *miR-223-3p*. Future prospective, multi-center studies are warranted to validate the generalizability of our conclusions using a more representative cohort. The single-center setting, while ensuring consistency in data collection and patient management, introduces inherent limitations. It restricted the sample size, which, for a heterogeneous condition like HBV-ACLF, limited our ability to perform meaningful subgroup analyses (e.g., stratified by etiology or complications) and reduced statistical power. This study focused on the 28-day prognosis, which represents the most critical window for HBV-ACLF [52–54]; however, the lack of longer-term follow-up data (e.g., 90 days) limits the evaluation of the prognostic value of *miR-223-3p* for intermediate-term survival. This should be explored in future studies with extended observation periods. Extensive studies support *U6*/*GAPDH* as reference genes for miRNA quantification in serum and cell injury models [55–57]. However, their stability may vary under certain pathological conditions. Although this study did not observe instability in *U6*/*GAPDH* expression, the incorporation of spike-in controls could further improve data accuracy and reliability. Additionally, there is a lack of functional in vivo data to corroborate our in vitro findings. An in vitro model cannot recapitulate the immensely complex immune microenvironment and multicellular crosstalk (e.g., between hepatocytes, Kupffer cells, and lymphocytes) present in HBV-ACLF in vivo. Therefore, our mechanistic insights remain preliminary. Following *miR-223-3p* modulation, quantitative analysis at multiple time points and Western blot analysis could provide a more accurate description of this regulatory process. Additionally, dynamic observation of cellular morphological changes during proliferation and apoptosis experiments will enhance the intuitiveness of the results. Subsequent in vivo functional gain- or loss-of-function experiments in appropriate HBV-ACLF animal models are essential to confirm the therapeutic potential and translational value of *miR-223-3p*. Although *miR-223-3p* shows therapeutic potential, as evidenced by animal studies [16, 58], significant challenges remain regarding its delivery system and target specificity, which may limit its clinical applicability. The upstream regulatory mechanisms responsible for the downregulation of *miR-223-3p* in HBV-ACLF have not yet been fully elucidated. Key aspects such as the potential involvement of inflammatory signaling pathways and epigenetic modifications remain to be explored.

## Conclusion

Taken together, this study indicates that serum *miR-223-3p* represents a promising biomarker for short-term prognosis in patients with HBV-ACLF. Moreover, *miR-223-3p* attenuated in vitro-induced hepatocellular injury, suggesting its potential as a therapeutic target for HBV-ACLF.

## Supplementary Information

Supplementary Material 1.

## Data Availability

No datasets were generated or analysed during the current study.

## Abbreviations

ACLF: Acute-on-chronic liver failure
ALSS: Artificial liver support system
ALT: Alanine aminotransferase
AST: Aspartate aminotransferase
FBS: Fetal bovine serum
HBV-ACLF: Hepatitis B virus-related acute-on-chronic liver failure
INR: International normalized ratio
MELD: Model for end-stage liver disease
miRNAs: MicroRNAs
MUT: Mutant
PLT: Platelet count
SCr: Serum creatinine
TBil: Total bilirubin
WBC: White blood cells
WT: Wild type
