# Supplementary material for: miR-223-3p predicts prognosis of hepatitis B virus-related acute-on-chronic liver failure and is involved in hepatocyte injury via HSP90B1
Source: Hereditas. 2025 Nov 29;163:6. doi: 10.1186/s41065-025-00610-5 (PMC12771722; doi:10.1186/s41065-025-00610-5)
Supplement: Supplementary file 1 — Supplementary Material 1. [file 41065_2025_610_MOESM1_ESM.docx]

**Table S1.** Primers used in qPCR.

| Name | Primer sequence (5'-3') |
| --- | --- |
| *MiR-223-3p* | Forward: GCGCGTGTCAGTTTGTCAAAT |
|  | Reverse: AGTGCAGGGTCCGAGGTATT |
| *U6* | Forward: GCTTCGGCAGCACATATACTAAAAT |
|  | Reverse: CGCTTCACGAATTTGCGTGTCAT |

**Table S2.** Subgroup analysis: association between *miR-223-3p* and overall survival in HBV-ACLF.

| Characteristic | HR factor | 95% CI | *P* value |
| --- | --- | --- | --- |
| Age |  |  |  |
| < 53 | 0.160 | 0.045-0.568 | 0.005 |
| > 53 | 0.173 | 0.050-0.598 | 0.006 |
| Gender |  |  |  |
| Male | 0.218 | 0.087-0.548 | 0.001 |
| Female | 0.017 | 0.001-0.298 | 0.005 |
| ACLF grade |  |  |  |
| I/II | 0.217 | 0.085-0.551 | 0.001 |
| III | 0.074 | 0.012-0.459 | 0.005 |

Abbreviations: HBV-ACLF, hepatitis B virus-acute-on-chronic liver failure; HR, hazard ratio; CI, confidence interval.

**
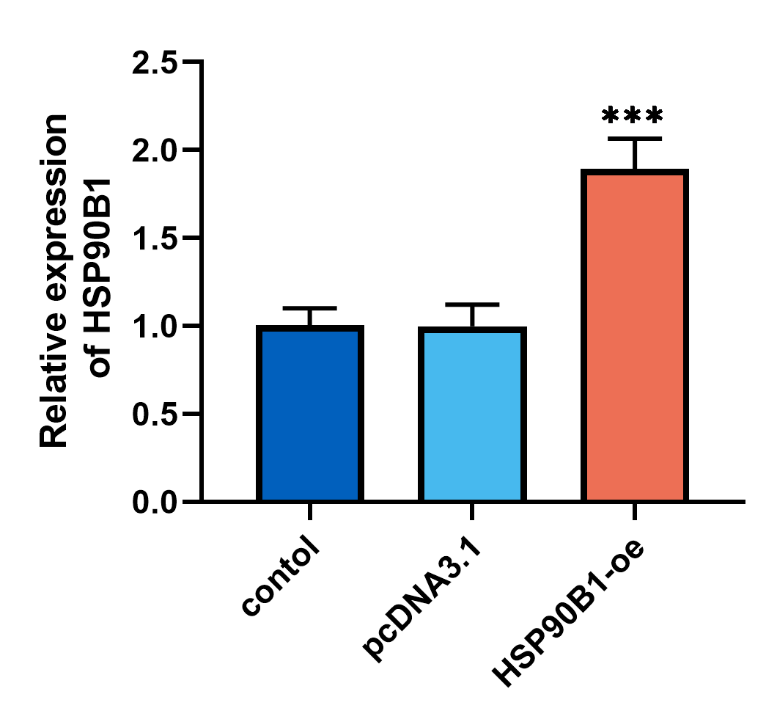
**

**Figure S1.** Transfection efficiency of the *HSP90B1* overexpression vector. (one-way ANOVA with Tukey’s test, ***P < 0.001).


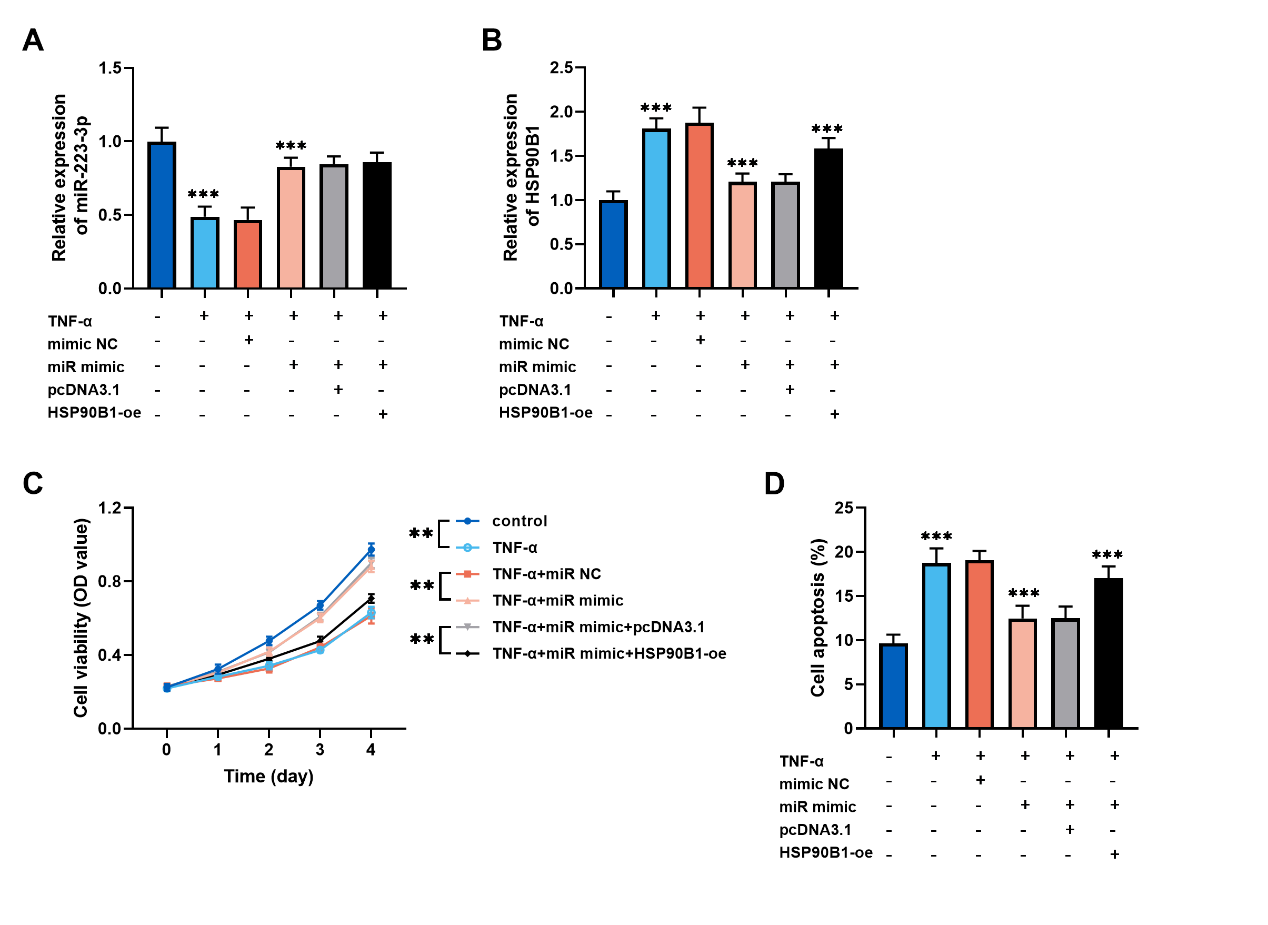


**Figure S2.** The biological functions of *miR-223-3p* were exerted through the regulation of *HSP90B1*. A. TNF-α downregulated *miR-223-3p* expression in MIHA cells, whereas transfection with *miR-223-3p* mimics restored its expression levels. B. In TNF-α-induced MIHA cells, *miR-223-3p* suppressed *HSP90B1* expression, whereas transfection of *HSP90B1*-oe restored its expression. C-D. In TNF-α-induced MIHA cells, overexpression of *HSP90B1* repressed the promotion of cell proliferation (C) and attenuated the inhibition of apoptosis (D) by *miR-223-3p*. (one-way or two-way ANOVA with Turkey’s test, **P < 0.01, ***P < 0.001).
